# Efficacy and safety of transcarotid compared to transfemoral approach of patent ductus arteriosus stenting in duct-dependent cyanotic heart disease

**DOI:** 10.1186/s43044-023-00371-4

**Published:** 2023-06-12

**Authors:** Made Satria Yudha Dewangga, Radityo Prakoso, Anna Ulfah Rahajoe, Oktavia Lilyasari

**Affiliations:** 1 Department of Cardiology and Vascular Medicine, National Cardiovascular Center Harapan Kita Jakarta, University of Indonesia, Jakarta, Indonesia; 2 Department of Cardiology and Vascular Medicine, Faculty of Medicine, Universitas Udayana-Prof. I.G.N.G Ngoerah Hospital, Bali, Indonesia

**Keywords:** Vascular access, Patent ductus arteriosus, Safety, Efficacy, Transcarotid, PDA stenting

## Abstract

**Background:**

Minimally invasive transcatheter approaches were usually done for patent ductus arteriosus (PDA) with duct-dependent pulmonary circulation. There are two ways to establish vascular access, by using transfemoral either femoral vein (FV) or femoral artery (FA) and transcarotid artery (CA) with surgical cutdown approach to access the PDA and then provide good support for the balloon and the stent to be safely deployed. This study aims to compare the efficacy and safety of transcarotid with surgical cutdown compared to the transfemoral approach of patent ductus arteriosus stenting in duct-dependent cyanotic heart disease.

**Results:**

Overall procedural complication rates were higher in the FA/FV approach than in the CA approach (51% vs. 30%). The incidence of acute limb ischemia in the FA approach is significantly higher than in the CA approach (*P* < 0.05). No acute thrombosis/occlusion of the carotid artery was assessed by carotid vascular ultrasound in 2-day series.

**Conclusions:**

The transcarotid approach with surgical cutdown may offer a secure and more efficient means of accessing the PDA, particularly for those emerging from the underside of the aortic arch.

## Highlights

- The CA approach may allow for successful stenting of the PDA if the anatomy and origin of the PDA are suitable.
- CA approach offers better control and trajectory to vertically oriented PDA.
- The CA approach is effective and efficient when PDA anatomy is favorable relative to shorter fluoroscopy time and procedural duration.

## Background

In some patients with cyanotic congenital heart disease (CCHD), a patent ductus arteriosus (PDA) is needed to maintain stable hemodynamics and increase blood oxygen saturation. These cardiac lesions are called duct-dependent CCHD, including pulmonary atresia (PA) with or without ventricular septal defect (VSD), transposition of great arteries with intact ventricular septum (TGA–IVS) or any intracardiac defect with severe/critical pulmonary stenosis (PS) [1].

The PDA in patients with duct-dependent pulmonary circulation may originate from the descending aorta, the underside of the aortic arch, the subclavian artery, the innominate artery, and the ascending aorta. A straight PDA was 8 times more likely to be successfully stented than a tortuous PDA. The definition of PDA "tortuosity" is mainly subjective, and most PDAs with higher degrees of tortuosity were seen arising from the underside of the arch [2]. Qureshi et al. used a ductal classification scheme based on the tortuosity of the duct and found that patients with the highest tortuosity had an increased need for PDA stent re-intervention and pulmonary arterioplasty [3].

With minimally invasive transcatheter approaches, ductus stent implantation has been shown as an effective and relatively safe alternative to surgical Blalock–Taussig (BT) shunt to establish the source of pulmonary blood flow. Nevertheless, the procedure can be technically challenging with a 16–20% failure rate and may lead to detrimental complications, such as major bleeding, stent thrombosis/restenosis, stent dislodgement, acute limb ischemia or ischemic stroke [4]. Correct vascular access site facilitates the procedure and minimizes the risk of complications. The PDA should be approached from the nearest and straightest vascular entry site. Access from the femoral artery is suitable for the PDA, which arises from the proximal descending aorta. The carotid or jugular artery approach is useful when the PDA arises from the underside of the aortic arch. Recent studies have shown that percutaneous carotid artery access has a low rate of vascular complications, with preserved vascular patency on follow-up and no neurological sequelae [5, 6].

There are two ways to establish vascular access, by using transfemoral either femoral vein (FV) or femoral artery (FA) and transcarotid artery (CA) approach to access the PDA and then provide good support for the balloon and the stent to be safely deployed. The Society of Cardiovascular Angiography and Interventions (SCAI) has recently developed ways to choose the preferred access site for PDA stenting. In general, arterial access with the straightest PDA access is favorable. Simple PDA anatomy is typically amenable to a transfemoral approach. Vertical or tortuous PDAs are better accessed from a trans-carotid approach, but there was no direct comparison regarding the efficacy and safety of these approaches [6, 7]. Therefore, this study is conducted to assess these two approaches' efficacy and safety aspects to support evidence for clinical strategies.

## Method

From the database, a retrospective analysis was performed on infants and children with ductal-dependent pulmonary blood flow who underwent PDA stenting between January 1, 2018, and May 31, 2022. We included all infants and children (< 5 years) with confluent pulmonary arteries and ductal-dependent pulmonary blood flow who underwent PDA stenting as a palliative treatment to provide a source of pulmonary blood flow or emergency procedure to increase peripheral oxygen saturation. The approach for PDA stenting was noted and codified as either (a) FV/FA or (b) CA.

**Fig. 1:**
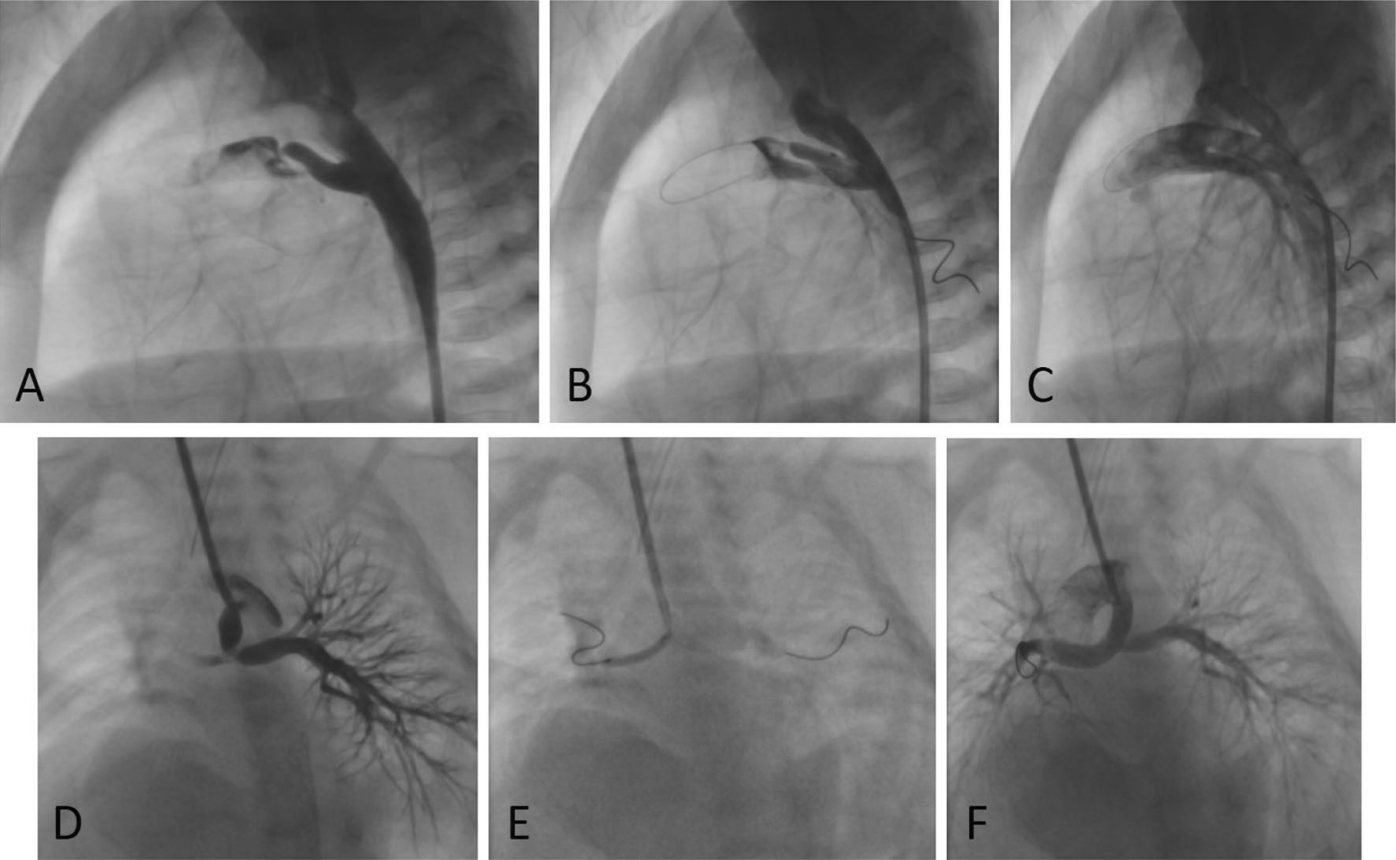
Various PDA morphology and origin in duct-dependent cyanotic congenital heart disease. **A**–**C** A relatively horizontal PDA with tight distal stenosis arising from descending aorta was successfully stented with FA approach in a PA–IVS patient; **D**–**F**: Vertical PDA from the underside of the aortic arch with trifurcation stenosis in a patient with PA-inlet VSD was successfully stented with CA approach

Carotid access was performed without ultrasound guidance and using the surgical arterial cutdown method done by the National Cardiovascular Centre Harapan Kita (NCCHK) vascular surgery team. A 5-F pediatric sheaths were inserted into the carotid artery after careful isolation. If the preprocedural echocardiography revealed the left aortic arch, the right common carotid artery was punctured; if the aortic arch is right-sided, the left common carotid artery was punctured. Femoral arterial access was performed with the standard, modified Seldinger technique and a 4/5-F slender sheath was utilized. A 3.5/5-F Judkins right guiding catheter was inserted to facilitate soft coronary wire, coronary balloon and the stent (Fig. 1).

Preprocedural transthoracic echocardiography (TTE) emphasizes high parasternal and suprasternal views done to decide vascular access and catheterization strategy. When PDA arises from the underside aortic arch and relatively have vertical direction, we are likely to establish CA access, and when PDA arises from descending aorta, we decide to establish FA/FV access instead.

Patients receive standard intravenous heparin of 50–100 IU/Kg during the procedure and continue for 3 days after the procedure unless uncontrolled bleeding occurs. Regarding antiplatelet therapy, we give a low dose of aspirin (5 mg/Kg) after the procedure to maintain stent patency. The role of clopidogrel remains unclear in ductal stenting, although some centers prefer dual therapy, especially if a drug-eluting stent is used [8]. All patients received a drug-eluting stent (DES) to decrease the risk of neointimal hyperplasia, thrombosis and restenosis in ductal stenting. For the choice of stent size, we follow the general agreement that the stent should be inflated to a diameter between 3 and 3.5 mm in patients weighing < 3 kg, between 3.5 and 4 mm in patients weighing 3–4 kg, and > 4.5 mm in patients weighing ≥ 4 kg [8].

The procedural time was counted from established access until the successful deployment of the PDA stent. Vascular access was examined with clinical evaluation and vascular ultrasound immediately after the procedure and the day after.

Patient demographics, cardiac anatomy, associated interventional procedures, and ultrasound results were obtained via retrospective record review. Continuous variables were expressed as mean ± standard deviation (SD) or median with interquartile ranges and were compared using the Student's *T*-test or Mann–Whitney U test as appropriate. Categorical data were presented as frequency (percentages) and were compared using Fisher's exact or chi-square test.

## Results

PDA stenting was performed on 76 patients, but only 61 patients had complete medical records. FA/FV approach was performed in 34 patients, and the CA approach was performed in 27 patients. There was one failed ductal cannulation in FA/FV approach in unplanned PDA stenting due to severe desaturation during angiography. It is because PDA was arising from the underside aortic arch, which is vertically oriented and highly tortuous. The procedure was aborted. Another failed case in FA/FV approach was in patients with hypoplastic left heart syndrome and mitral atresia with interrupted aortic arch. If the patient suffered from severe desaturation and progressive lactic acidosis during the procedure, we decided to abort the procedure.

Descriptions of clinical and anatomic characteristics of CA and FA/FV groups are summarized in Table 1. There are no significant differences between the two groups regarding patients' sex, age, prematurity, weight, genetic syndrome, periprocedural inotropic usage, and aortopulmonary collaterals. Prostaglandin E1 infusion and other medical conditions, including sepsis and anemia preprocedural, are more common in patients with the CA approach than in the FV/FA approach (Fig. 1).

**Table 1:** Clinical and anatomic characteristics by access site

Characteristics	FV/FA (*n* = 34)	CA (*n* = 27)	*P*
Sex, *n* (%)			
Male	16 (47)	11 (40)	0.408
Female	18 (53)	17 (60)	
Age at PDA stenting, days (median: min–max)	63: 7–485	42:11–1490	0.649
Weight at procedure, Kgs (mean: min–max)	4.5: 3–8.3	3.9: 2.1–7.4	0.1
Primary Diagnostic Category, *n* (%)			
PA–IVS	19 (56)	5 (15)	0.006*
PA with VSD/CAVSD	3 (9)	21 (62)	< 0.001*
TGA–IVS	5 (15)	0 (0)	0.038
TGA with restrictive VSD	3 (9)	0 (0)	0.166
DORV with PS	2 (6)	1 (3)	0.693
PDA as single source of PBF, *n* (%)	22 (65)	24 (70)	0.17
Krichenko PDA Classification, *n* (%)			
Type A	24 (70)	14 (41)	0.355
Type B	1 (3)	2 (7)	0.423
Type C	12 (11)	2 (7)	0.453
Type D	2 (6)	5 (15)	0.129
Type E	3 (9)	2 (7)	0.61
Significant Branch PA stenosis	3 (9)	4 (12)	0.241
PGE-1 usage pre-intervention, *n* (%)	3 (16)	10 (37)	0.002*
Inotropic administration pre-intervention, *n* (%)	10 (29)	7 (26)	0.079
Other medical conditions, *n* (%)			
Sepsis	2 (6)	2 (7)	0.089
Anemia preprocedural	4 (12)	2 (7)	0.633
Others	0 (0)	2 (7)	0.599
Location of the PDA, *n* (%)			
Underside Aortic Arch	3 (9)	25 (93)	< 0.001*
Descending Aorta	29 (85)	2 (7)	< 0.001*
Aborted left/right aortic arch	2 (6)	0 (0)	0.29
Other interventions, *n* (%)			
BAS	16 (47)	2 (7)	0
IAS stenting	1 (3)	0 (0)	0.557
TPM	1 (3)	0 (0)	0.557
MAPCAs embolization	0 (0)	2 (7)	0.192

*(*p* < 0.05)

*BAS* balloon atrial septostomy, *CAVSD* complete atrio-ventricular septal defect, *DORV* double outlet right ventricle, *IAS* interatrial septum, *ICU* Intensive Care Unit, *IVS* intact ventricular septum, *MAPCA* major aorto-pulmonary collateral arteries, *PA* pulmonary atresia, *PBF* pulmonary blood flow, *PDA* patent ductus arteriosus, *TGA* transposition of great arteries, *TPM* temporary pacemaker, *VSD* ventricular septal defect

Transfemoral approach was preferred (85%) in cases with PDA from proximal descending aorta. In contrast, CA was preferred (93%) in cases with tortuous PDA arising from under the surface of the aortic arch. Associated interventions (BAS, IAS stenting) prior to the PDA stent procedure were more common among the FA/FV approach (47%) than the CA approach (7%), and this is due to hemodynamic of pulmonary atresia with the intact ventricular septum (PA–IVS) that required adequate mixing in interatrial level.

The average fluoroscopy and procedural time in the CA approach were shorter than FV/FA approach, although not statistically significant (12.9 vs. 9.4 min) and (48 vs. 75 min), respectively. There was no significant difference in the average ICU length of stay between the two groups (5.3 vs 4.7 days).

Procedural and postprocedural complications were analyzed and are summarized in Table 2. Overall procedural complication rates were higher in the FA/FV approach than in the CA approach (51% vs 30%). The incidence of acute limb ischemia in the FA approach is significantly higher than in the CA approach (*P* < 0.05). No acute thrombosis/occlusion of the carotid artery was assessed by carotid vascular ultrasound in 2-day series. Acute limb ischemia was successfully treated with unfractionated heparin infusion and resolved completely. The two groups had no significant differences in complications (hemodynamic instability, major site bleeding, stent dislodgement, stent restenosis, and arrhythmias) (Table 3).

**Table 2:** Short-term procedural outcomes

Outcome parameter	FA/FV, *n* (%)	CA, *n* (%)	*P* value
Successful procedures	32 (94)	27 (100)	0.633
Fluoroscopy time, mins (median: min–max)	12.9: 2–33	9.4: 2–36	0.105
Procedural time, mins (median: min–max)	64.79: 11–130	44.28: 15–82	0.105
ICU length of stay, days (median: min–max)	5: 2–17	5: 2–18	0.745

**Table 3:** Complications related to vascular access

Complications	FA/FV, *n* (%)	CA, *n* (%)	*P* value
Site bleeding requiring transfusion	7 (21)	7 (26)	0.531
Stent restenosis	1 (3)	1 (4)	0.443
Acute vascular site occlusion	5 (15)	0 (0)	0.047
Stent dislodgement	1 (3)	0 (0)	0.557
Significant arrhythmia	1 (3)	0 (0)	0.557
Failed cannulation	1 (3)	0 (0)	0.307

One urgent transcutaneous pacemaker (TPM) insertion was performed in TAVB with an FV approach in PA with a complete atrioventricular septal defect (CAVSD) patient. This is probably due to catheter manipulation in the right ventricle to reach the aorta, and the access was switched to FA. One case of stent dislodgement in the FV approach in a patient with PA–VSD and PDA arose from an aborted left aortic arch. Tight stenosis and relatively sharp angulation made it difficult to cross the stent into PDA using *'the naked technique*’ 6-mm vascular stent, and eventually, the stent was dislodged to the inferior vena cava during the pullback maneuver. The dislodged stent was retrieved with a hybrid procedure, surgically opening the right atrium, and the stent was retrieved with snaring from the right atrial roof. After snaring, the procedure was continued with different methods (using guiding catheter and 4-mm coronary stent) and was successful.

## Discussion

**Fig. 2:**
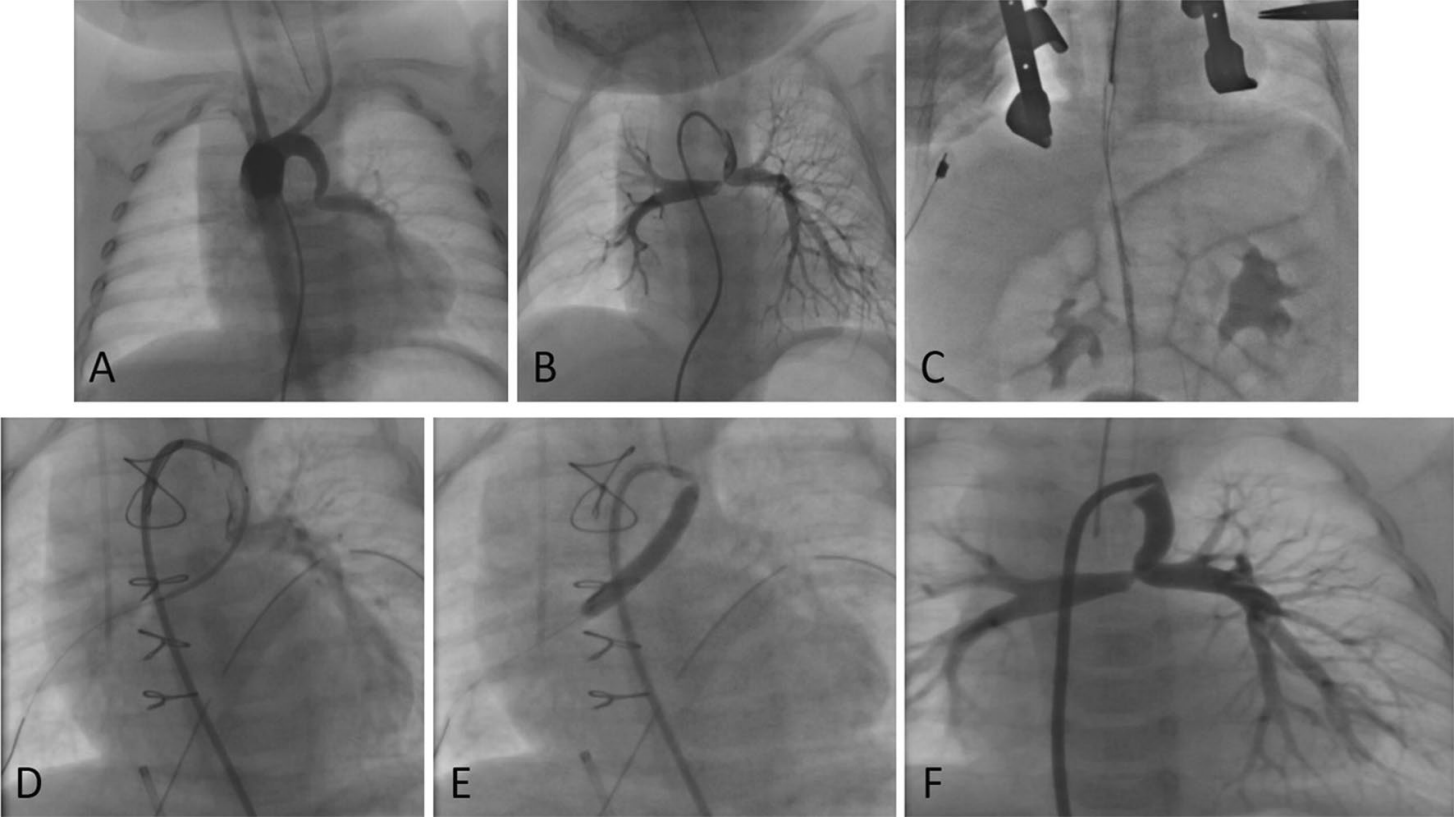
PDA stenting of a 3-month-old baby with pulmonary atresia, subaortic VSD with vertical PDA arising from left aborted aortic arch accessed from FV. **A**, **B** vertically oriented PDA from left aborted aortic arch; **C** A 6-mm vascular stent failed to cross the PDA and was embolized to IVC when retracted back to the sheath; **D**, **E** A 4 mm coronary stent was successfully inserted through the PDA; **F** the final result showed opened PDA to the left pulmonary artery

It is known that the CA approach was more common for PDAs originating from the underside of the aortic arch and for PDAs that were more tortuous. PDA anatomical characteristics requiring CA access are likely associated with the diagnosis of pulmonary atresia with ventricular septal defect (PA–VSD) (62%). Whereas in FA, access is more likely to be with the diagnosis of PA–IVS (56%) and TGA–IVS (24%). Complex PDA anatomy (type D and E) is more likely to be encountered in the CA approach (23%) than in the FA approach (15%) (Fig. 2). The CA and the FV/FA had similar procedural complication rates. Our findings suggest that the CA approach may allow for successful stenting of PDA if the anatomy and origin of the PDA are suitable. In our center, all carotid artery approaches are established by surgical cutdown, allowing better visualization of the carotid artery.

In our center, CA or FA/FV approaches have similar high procedural success rates due to careful preprocedural echocardiography assessment to determine what access should be taken. If echocardiography visualization is not good enough and we doubt the PDA anatomy, a computed tomography scan would add value in outlining ductal origin, insertion, size, course and curvature [9].

Although not statistically significant, a relatively shorter average fluoroscopy and procedural time were achieved in the CA approach than FA/FV approach. We follow the Society of Cardiovascular Angiography and Interventions (SCAI) recommendation to perform the CA approach when the PDA arose from the underside of the arch and if there was vertical and tortuous (type D or E PDA by Krichenko classification). On the other hand, most of the procedures from the FA were for type A with relatively straight PDAs arising from the descending aorta. CA approach offers better control and trajectory to vertically oriented PDA. Access from the CA may provide more stable access for maintaining the wire position and increasing the procedure's safety profile. The relatively shorter fluoroscopy time and procedural duration in the CA approach also proved that the CA approach is effective and efficient when PDA anatomy is favourable. [1, 8, 9]

There were concerns about major bleeding when we performed surgical cutdown and the increasing need for blood transfusion. Nevertheless, our study suggests no significant differences in major bleeding compared to FV/FA approach. This is consistent with previous findings by Bauser-Heaton et al. that the CA approach is associated with a similar procedural burden with FV/FA.

There was one failed PDA cannulation in FA/FV approach due to severe proximal PDA stenosis arising from the aortic arch's underside. This patient originally planned for diagnostic catheterization, but suddenly desaturated and supraventricular tachycardia occurred during angiography; therefore urgent PDA stenting procedure was initialized. Stenosis of the aortic end of the PDA should be carefully assessed for the feasibility of any approach.

Previous findings by Justino and Petit reported that percutaneous CA access for neonatal intervention was associated with low rates of vascular complications. Their report included 47 procedures from the carotid approach (23 of which were for PDA stenting), and 3 (6.4%) procedures was associated with carotid thrombosis, all resolved completely. The sole case of complete and persistent occlusion was with surgical CA cutdown and primary surgical repair. This surgical outcome is consistent with limited reports suggesting a significant rate (31–33%) of severe stenosis or occlusion following surgical CA cutdown in children [1]. However, this study demonstrates the feasibility and safety of percutaneous PDA stenting from the surgical-cutdown CA approach. Specifically, we observed no acute carotid artery occlusion in the CA group assessed with carotid vascular ultrasound 24 h after the procedure. We performed surgical cutdown due to concerns about the potential neurovascular sequelae in the percutaneous approach and decreasing the probability of false route puncture. We noted that careful surgical cutdown by an experienced surgery team could minimize vascular injury or trauma during the procedure. Whether or not surgical-cutdown CA access is safer than the percutaneous approach for these procedures cannot be determined from our study [1].

Acute femoral artery occlusion in the FA approach occurred in some patients, possibly related to the relatively large sheath used to facilitate stent deployment: The larger the sheath, the more probability of damaging the vascular wall and creating thrombus formation. Glatz et al. reported that 44% of neonates < 4 kg were found to have an acute arterial injury when the FA was accessed.

### Study limitation

This study is only retrospectively based on one center, so it has a less generalized picture of the population.

## Conclusions

This study demonstrates the efficacy and safety of PDA stenting with the CA approach, especially for vertically oriented and anatomically complex PDA anatomies. The most common complications of CA access are site bleeding associated with a blood transfusion but not caused hemodynamic compromise. CA approach with surgical cutdown is relatively safe from vascular thrombosis compared to the FA approach, consistent with previous reports.

## Data Availability

The datasets used and/or analyzed during the current study are available from the corresponding author on reasonable request.

## Abbreviations

BT: Blalock–Taussig
CA: Carotid artery
CAVSD: Complete atrioventricular septal defect
CCHD: Cyanotic congenital heart disease
DES: Drug-eluting stent
FA: Femoral artery
FV: Femoral vein
NCCHK: National Cardiovascular Centre Harapan Kita
PA: Pulmonary atresia
PA–VSD: Pulmonary atresia with ventricular septal defect
PDA: Patent ductus arteriosus
PS: Pulmonary stenosis
SCAI: Society of Cardiovascular Angiography and Interventions
SD: Standard deviation
TGA–IVS: Transposition of great arteries with intact ventricular septum
TPM: Transcutaneous pacemaker
TTE: Transthoracic echocardiography
VSD: Ventricular septal defect
